# A multimodal marker for cognitive functioning in multiple sclerosis: the role of NfL, GFAP and conventional MRI in predicting cognitive functioning in a prospective clinical cohort

**DOI:** 10.1007/s00415-023-11676-4

**Published:** 2023-04-27

**Authors:** Maureen van Dam, Brigit A. de Jong, Eline A. J. Willemse, Ilse M. Nauta, Marijn Huiskamp, Martin Klein, Bastiaan Moraal, Sanne de Geus-Driessen, Jeroen J. G. Geurts, Bernard M. J. Uitdehaag, Charlotte E. Teunissen, Hanneke E. Hulst

**Affiliations:** 1 MS Center Amsterdam, Anatomy and Neurosciences, Vrije Universiteit Amsterdam, Amsterdam Neuroscience, Amsterdam UMC Location VUmc, Amsterdam, The Netherlands; 2 MS Center Amsterdam, Neurology, Vrije Universiteit Amsterdam, Amsterdam Neuroscience, Amsterdam UMC Location VUmc, Amsterdam, The Netherlands; 3 Neurochemistry Lab, Department of Clinical Chemistry, Amsterdam UMC, Vrije Universiteit Amsterdam, Amsterdam Neuroscience, De Boelelaan 1117, Amsterdam, The Netherlands; 4 Neurology Clinic and Policlinic, Departments of Head, Spine and Neuromedicine, Biomedicine and Clinical Research, Research Center for Clinical Neuroimmunology and Neuroscience (RC2NB), University Hospital Basel, University of Basel, Basel, Switzerland; 5 Department of Medical Psychology, Amsterdam UMC, Vrije Universiteit Amsterdam, Amsterdam Neuroscience, De Boelelaan 1117, Amsterdam, The Netherlands; 6 Department of Radiology and Nuclear Medicine, MS Center Amsterdam, Amsterdam UMC, Vrije Universiteit Amsterdam, Amsterdam Neuroscience, De Boelelaan 1117, Amsterdam, The Netherlands; 7 Institute of Psychology, Health, Medical and Neuropsychology Unit, Leiden University, Wassenaarseweg 52, Leiden, The Netherlands

**Keywords:** Multiple sclerosis, Cognition, MRI, Neurofilament light, GFAP, Serum, CSF

## Abstract

**Background:**

Cognitive impairment in people with MS (PwMS) has primarily been investigated using conventional imaging markers or fluid biomarkers of neurodegeneration separately. However, the single use of these markers do only partially explain the large heterogeneity found in PwMS.

**Objective:**

To investigate the use of multimodal (bio)markers: i.e., serum and cerebrospinal fluid (CSF) levels of neurofilament light chain (NfL) and glial fibrillary acidic protein (GFAP) and conventional imaging markers in predicting cognitive functioning in PwMS.

**Methods:**

Eighty-two PwMS (56 females, disease duration = 14 ± 9 years) underwent neuropsychological and neurological examination, structural magnetic resonance imaging, blood sampling and lumbar puncture. PwMS were classified as cognitively impaired (CI) if scoring ≥ 1.5SD below normative scores on ≥ 20% of test scores. Otherwise, PwMS were defined as cognitively preserved (CP). Association between fluid and imaging (bio)markers were investigated, as well as binary logistics regression to predict cognitive status. Finally, a multimodal marker was calculated using statistically important predictors of cognitive status.

**Results:**

Only higher NfL levels (in serum and CSF) correlated with worse processing speed (*r* = − 0.286, *p* = 0.012 and *r* = − 0.364, *p* = 0.007, respectively). sNfL added unique variance in the prediction of cognitive status on top of grey matter volume (NGMV), *p* = 0.002). A multimodal marker of NGMV and sNfL yielded most promising results in predicting cognitive status (sensitivity = 85%, specificity = 58%).

**Conclusion:**

Fluid and imaging (bio)markers reflect different aspects of neurodegeneration and cannot be used interchangeably as markers for cognitive functioning in PwMS. The use of a multimodal marker, i.e., the combination of grey matter volume and sNfL, seems most promising for detecting cognitive deficits in MS.

**Supplementary Information:**

The online version contains supplementary material available at 10.1007/s00415-023-11676-4.

## Introduction

Cognitive impairment is one of the most disabling symptoms of multiple sclerosis (MS), significantly hampering day-to-day functioning [1]. In an effort to monitor cognitive functioning in MS, understanding its underlying neurobiological correlates is of utmost importance. To date, most studies investigated magnetic resonance imaging (MRI) characteristics in relation to cognitive performance, which has taught us that cognitive impairment is associated with neurodegeneration such as cortical and deep grey matter atrophy [2, 3], as well as with functional impairment of neuronal networks [4]. However, the clinical implementation of these prognostic biomarkers is limited, as these markers cannot fully account for the large heterogeneity found between people with MS (PwMS) [5]. The complex pathology of MS, including inflammation, demyelination and neurodegeneration warrants a multimodal biomarker linking both molecular and imaging biomarkers [6].

Recent studies focused on the combination of neurofilament light chain levels in serum (sNfL) and conventional imaging markers (e.g., lesion load and grey matter volume) for predicting cognitive functioning in PwMS [7, 8]. NfL reflects the major intermediate cytoskeletal protein of axons and is considered to be a marker for neuro-axonal damage [9]. Indeed, increased levels of NfL in the serum and cerebrospinal fluid (CSF) of PwMS have been related to cognitive impairment [10] and decreased performance on multiple cognitive domains [7, 11], in various disease stages [8, 12], showing promising predictive value over time [10]. Another potentially interesting biomarker for the assessment of neurodegeneration in MS is glial fibrillary acidic protein (GFAP), the intermediate cytoskeletal protein of astrocytes [13, 14]. Both serum and CSF levels of GFAP have been shown to relate to disease type (i.e., increased levels of GFAP in progressive PwMS [15]) and disease severity (i.e., increased GFAP levels were associated with higher physical disability and longer disease durations [13, 16]). However, the association between GFAP and cognitive functioning in MS has yet to be established.

The aim of current study was to compare, confirm and combine (bio)markers of neurodegeneration (i.e., serum and CSF levels of both NfL and GFAP and conventional imaging markers) for its role in cognition in a clinical sample of PwMS that visited our outpatient clinic because of perceived cognitive complaints.

## Materials and methods

### Study population

**Fig. 1:**
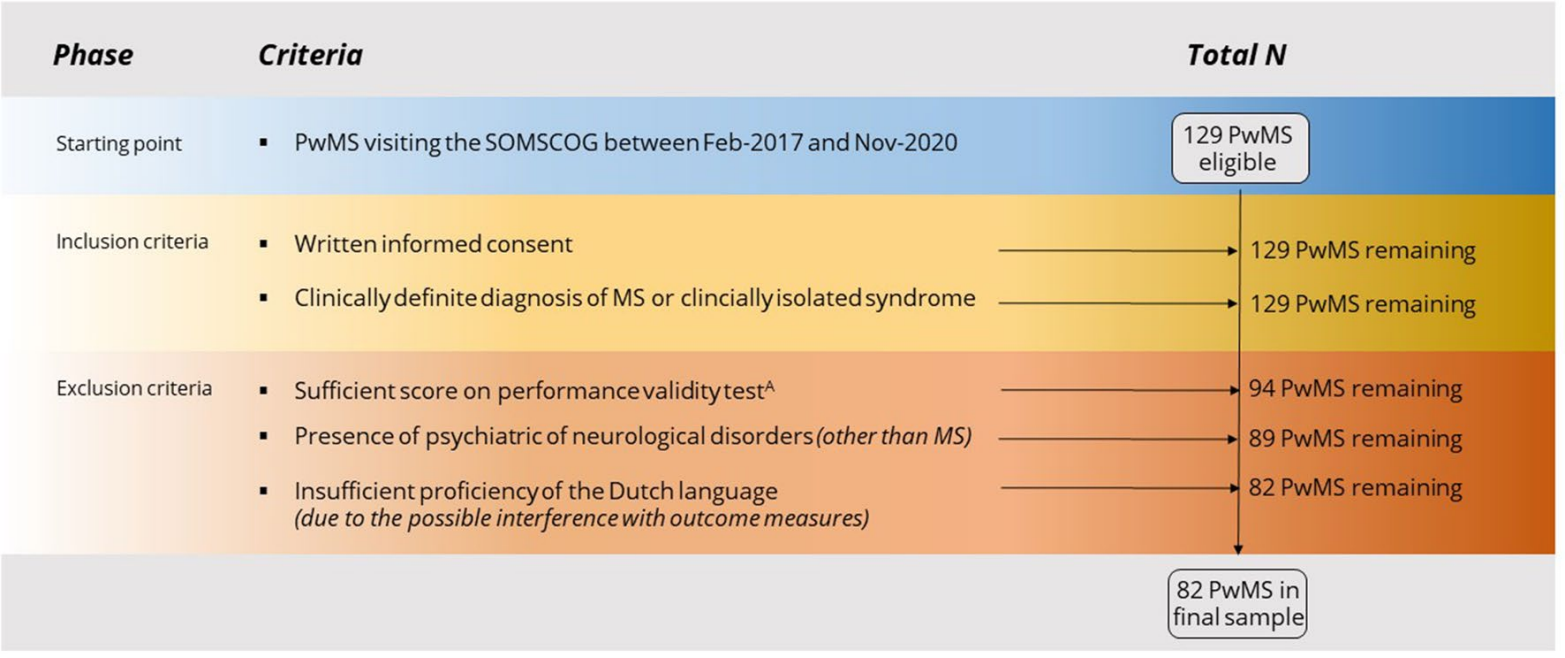
An overview of the included PwMS after applying the in- and exclusion criteria. ^A^PwMS were only included if they underwent a performance validity test (Amsterdam Short-Term Memory test) and had reached a sufficient score on this test. *PwMS* People with MS, *SOMSCOG* Second Opinion Multiple Sclerosis and Cognition

In total, 129 PwMS that visited the Second Opinion Multiple Sclerosis and Cognition (SOMSCOG) outpatient clinic of the MS Center Amsterdam between February 2017 and November 2020 (82 females, mean age: 48 ± 11 years) were included in this cross-sectional study. Individuals were referred to our SOMSCOG outpatient clinic because of cognitive complaints by their general physician or neurologist, and underwent an extensive diagnostic workup for cognitive impairment, including neuropsychological and neurological examination, MRI, blood sampling and lumbar puncture (all administered on the same day). PwMS were included if they gave written informed consent and had a clinically definite diagnosis of MS according to the McDonald MS criteria (2017–revised) [17] or clinically isolated syndrome. Additionally, PwMS were only included if they underwent a performance validity test (Amsterdam Short-Term Memory (ASTM) test [18, 19]) and had reached a sufficient score on this test, resulting in the exclusion of 35 PwMS. After applying the in- and exclusion criteria, a total of 82 PwMS remained eligible for data-analysis (Fig. 1). This is the second paper including data of this cohort, the first paper focused on performance validity [19].

### Demographics and clinical functioning

The demographic characteristics included age, sex and level of education (coded according the Verhage classification, a Dutch classification system for education [20]). Information on MS type, disease duration, and disease-modifying therapy (DMT; yes/no and if yes, first-line or second-line DMT) was collected from the medical charts. The level of physical disability was assessed by a certified examiner using the Expanded Disability Status Scale (EDSS) [21].

### Neuropsychological examination

Cognitive functioning was measured using an Dutch adaptation of the MACFIMS [22], consisting of the following five (sub)-domains: processing speed (Symbol Digit Modalities Test [23] and Stroop Color-Word Test cards I and II [24]), verbal memory (Dutch version of the California Verbal Learning Test version 2 [25]), visuospatial memory (Brief Visuospatial Memory Test—Revised [26]), executive function (EF)-verbal fluency (Controlled Oral Word Association Test [27]) and EF-inhibition (Stroop Color-Word Test Interference score [24]). The neuropsychological examination was administered by a certified clinical neuropsychologist at the department of Medical Psychology of the hospital.

Cognitive test scores were corrected for age, sex and educational level (if predictor was below *α* = 0.1 in regression analysis [28]), and transformed into five domain-specific Z-scores, based on a normative sample of Dutch healthy controls (*N* = 407). PwMS were classified as cognitively impaired (CI) if ≥ 20% of the cognitive test scores were ≥ 1.5SD below normative scores (corresponding to ≥ 3 out of 11 test scores), or otherwise as cognitively preserved (CP) [29].

Since multiple psychological factors are known for its impact on cognition, henceforth referred to as patient-reported outcome measures (PROMS), current study protocol included: self-perceived cognitive problems (using the MS Neuropsychological Questionnaire–Patient version, MSNQ-P [30]), symptoms of anxiety and depression (using the Hospital Anxiety and Depression Scale, HADS [31]), levels of fatigue (using the Checklist Individual Strength-20 Revised, CIS20-R [32]), and sleep-related problems (using the Athens Insomnia Scale, AIS [33]). For all aforementioned questionnaires, higher scores indicate more symptoms.

### Imaging markers

Seventy-eight PwMS (~ 91%) underwent MR scanning on a 3-Tesla whole-body scanner (General Electric Signa-HDxt, Milwaukee, WI, USA), with an 8-channel head coil (see the Supplemental Methods for the detailed MRI protocol). White matter lesions on FLAIR images were segmented after which lesions on T1-weighted images were filled using an automated lesion-filling technique (LEAP) [34]. The SIENAX pipeline was used to obtain estimates of global white matter and grey matter volumes. FIRST was then applied for the automatic segmentation and calculation of bilateral hippocampus and thalamus volumes, areas known to be associated with cognitive decline in MS. All volumes were corrected for head size using the V-scaling factor obtained by SIENAX.

### Fluid biomarkers

Blood samples were collected in serum tubes through venipuncture for 78 PwMS (~ 95%), whereas CSF samples were obtained by lumbar puncture for 54 PwMS (~ 66%; see Supplemental Methods) [35]. Serum and CSF NfL and GFAP levels were quantified in parallel on Single Molecule Array (Simoa) HD-1 analyzers (Quanterix) using the Simoa NF-light Advantage Kit (Quanterix) and the Simoa GFAP Discovery Kit (Quanterix) [36]. Paired CSF and serum samples per PwMS were analyzed within one run. The average intra-assay coefficient of variation (CV) of sample duplicates was 5.3 ± 4.1% for serum GFAP (sGFAP) and 4.1 ± 3.7% for CSF GFAP (cGFAP); NfL measurements were performed in singlicates. One serum NfL (sNfL) measure failed due to a debris error (total *N* sNfL = 77). For sGFAP, two samples were measured in singlicate and for cGFAP, one sample was measured in singlicate.

### Statistical analysis

Normality of variables was explored using the Shapiro–Wilk test and histogram inspection of the residuals. In case of non-normally distributed data, logarithmic (for NLV, sNfL and CSF NfL (cNfL)) or square root transformations (for disease duration, sGFAP and cGFAP) were applied. Fluid and imaging (bio)markers were corrected, regression based, for sex and age (if *p* value of demographic variable was smaller than *α* = 0.1 in the model). The following correction formulas were applied:

$$\mathrm{sNfL } \, \left(\mathrm{corrected}\right)=\mathrm{ LG}10\left(\mathrm{sNfL}\right)- 0.009 \times \mathrm{ age } \, \left(\mathrm{in years}\right).$$

$$\mathrm{NGMV }(\mathrm{corrected}) = \, \mathrm{ NGMV }+ 2.622 \times \mathrm{ age } \, (\mathrm{in years}) - 46.680 \times \mathrm{ sex }(0 =\mathrm{ female}).$$

To investigate differences between CP and CI, independent samples *t-*tests were applied (for age, disease duration, PROMS, corrected fluid and imaging (bio)markers), or a Mann–Whitney *U* test for EDSS. Chi-square tests were used to investigate CP vs. CI differences in sex, educational level, type of MS, use of DMT and type of DMT (first-line vs. second-line). Pearson correlations were used to investigate the association (1) between corrected fluid biomarkers and cognitive domains, (2) between corrected imaging markers and cognitive domains and (3) between corrected fluid and imaging (bio)markers. Outcomes were Bonferroni corrected: the *α*-level of 0.05 was divided by the number of fluid (*p* < 0.0125) or imaging (bio)markers (*p* < 0.01).

Two binary logistic regression models (using either serum or CSF markers) with forward selection were run to identify the predictors of cognitive status. The choice of running two separate regressions was made as a significant part of the sample did not receive the lumbar puncture. To reduce the number of variables in the prediction models, the imaging markers were only inserted as predictor if significant group differences were present.

In a post-hoc analysis, the predictive value of the predictors alone was explored, while a weighted composite score of the significant predictors was calculated using the standardized betas. The composite score was further evaluated by drawing receiver operator characteristic curves and calculating the areas under the curves (AUCs) to determine diagnostic accuracy. Interpretation of the AUC was as follows: an AUC of 0.60 and 0.70 could be considered ‘poor’, an AUC of 0.70 to 0.80 could be considered ‘acceptable’ or ‘fair’, an AUC of 0.80 to 0.90 could be considered ‘good’ and an AUC above 0.9 could be considered ‘excellent’ [37]. Significance level was set at *α*-level < 0.05 and the statistical analyses were performed in SPSS 28.0 (IBM, Armonk, NY, USA).

## Results

### Study population

The final sample consisted of 82 PwMS (56 females, mean age = 47 ± 9 years, mean disease duration = 14 ± 9 years). Information on demographics, disease related variables, PROMS and imaging and fluid biomarkers can be found in Table 1. In 75% of the PwMS, the MSNQ-P was above the threshold of 27 [38], indicating the presence of self-perceived cognitive problems at the time of the visit. Performance on individual cognitive tests is included in Supplementary Table 2.

**Table 1:** Information on demographics, disease related variables, patient-reported outcome measures, imaging markers (in ml) and fluid biomarkers (in pg/ml) displayed for cognitive groups

	CP (*N* = 36)	CI (*N* = 46)	*p* value
Demographics			
Sex (female: male)	30: 6	26: 20	0.010*
Age	47.36 ± 9.95	47.07 ± 9.00	0.888
Educational level	6 [5–6]	6 [5–6]	0.913
Clinical functioning			
Disease duration^a^	13.04 ± 9.27	13.93 ± 8.92	0.693
EDSS	3.5 [2.5–4.0]	4.0 [3.0–4.5]	0.012*
MS type (CIS/RRMS/PPMS/SPMS/UN)	(2/26/4/4/0)	(2/28/3/12/1)	0.398
Use of DMT (yes: no)	18: 18	21: 25	0.696
Type of DMT (first-line: second-line)	13: 5	15: 10	0.407
Patient-reported outcome measures			
HADS anxiety	8.43 ± 4.13	8.73 ± 4.58	0.765
HADS depression	6.40 ± 4.09	7.64 ± 4.23	0.194
CIS20-R (fatigue)	90.21 ± 21.82	94.86 ± 17.20	0.295
MSNQ-P (cognitive complaints)	32.77 ± 10.28	33.53 ± 8.56	0.738
AIS (sleep-related problems)	6.80 ± 4.63	8.22 ± 4.60	0.175
Imaging markers (ml)^b^			
NGMV	805.03 ± 63.30	756.45 ± 59.89	0.002*
NWMV	684.12 ± 46.41	666.98 ± 48.43	0.141
NLV^c^	22.60 ± 20.38	35.27 ± 27.09	0.010*
Hippocampi	9.35 ± 1.11	8.57 ± 1.34	0.051
Thalami	19.28 ± 2.25	17.18 ± 2.76	0.002*
Fluid biomarkers (pg/ml)			
sNfL^c,d^	8.45 [5.19–12.67]	10.54 [8.41–15.25]	0.010*
sGFAP^a,e^	103.37 [79.59–147.88]	129.52 [92.57–184.89]	0.035*
cNfL^c,f^	561.11 [342.63–739.91]	579.74 [502.50–1109.46]	0.267
cGFAP^a,f^	7467.53 [5452.89–9223.88]	8039.92 [6818.75–9307.84]	0.073

Displayed are the mean and standard deviation of continuous variables, the median and interquartile range of ordinal or non-normally distributed data. Imaging markers and fluid biomarkers were corrected for age and sex (if appropriate) before tested

*p < 0.05

*CP* cognitively preserved, *CI* cognitively impaired, *EDSS* Expanded Disability Status Scale, *CIS* clinically isolated syndrome, *RRMS* relapsing remitting MS, *PPMS* primary progressive MS, *SPMS* secondary progressive MS, *UN* Unknown, *DMT* disease-modifying therapy, *HADS* Hospital Anxiety and Depression Scale, *CIS20-R* Checklist Individual Strength 20-Revised, *MSNQ M*S Neuropsychological Questionnaire, *AIS* Athens Insomnia Scale, *NGMV* normalized grey matter volume, *NWMV* normalized white matter volume, *NLV* normalized lesion volume, *sNfL* serum neurofilament light (NfL), *sGFA*P serum glial fibrillary acidic protein (GFAP), *cNfL* CSF NfL, *cGFAP* CSF GFAP

^a^Variable was square root-transformed before tested

^b^*N* = 78 (*N* CP = 36, *N* CI = 42). All volumes were normalized using the V-scaling factor

^c^Variable was log-transformed before tested

^d^*N* = 77 (*N* CP = 33, *N* CI = 44)

^e^*N* = 78 (*N* CP = 33, *N* CI = 45)

^f^*N* = 54 (*N* CP = 23, *N* CI = 31)

### Differences between cognitive groups

**Fig. 2:**
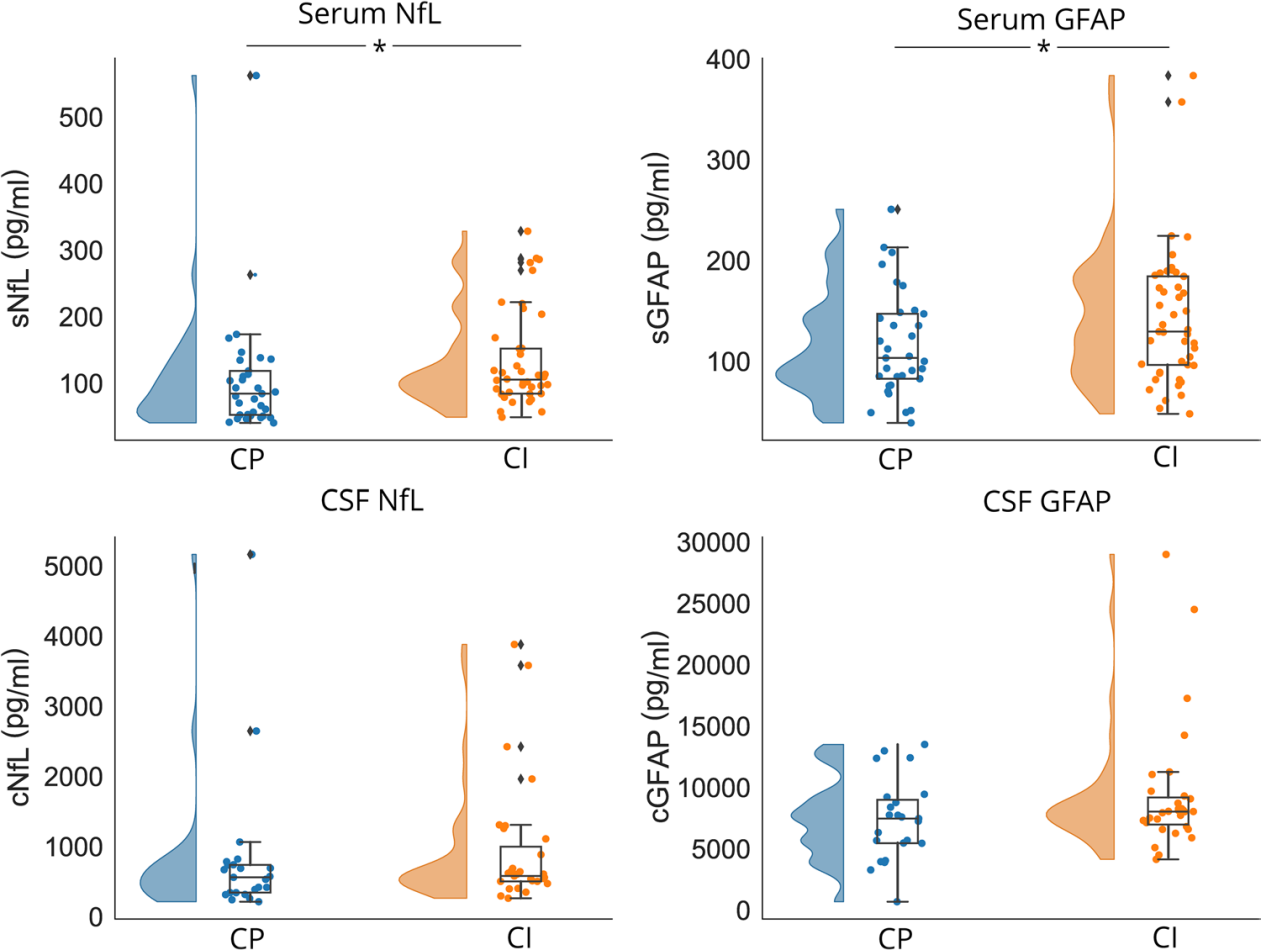
Differences in fluid biomarkers between cognitively preserved (CP) and cognitively impaired (CI) PwMS. Results indicate that sNfL and sGFAP are increased in CI PwMS, compared to CP PwMS (*p < 0.05). For illustrative purposes, the raw (non-transformed, not corrected) values of fluid biomarkers are shown. *PwMS* people with MS, *CP* cognitively preserved, *CI* cognitively impaired, *sNfL* serum neurofilament light (NfL), *sGFAP* serum glial fibrillary acidic protein (GFAP), *cNfL* CSF NfL, *cGFAP* CSF GFAP

An overview of the differences between groups can be found in Table 1 (in Supplementary Table 3 group differences with only complete data is included). Compared to CP PwMS (*N* = 36), the group of CI PwMS (*N* = 46) had worse physical disability (*p* = 0.012) and consisted of more men (16.7% versus 43.5%; *p* = 0.010). PROMS results were similar for both groups. CI PwMS had lower NGMV (*p* = 0.002, *d* = 0.741, 95% confidence interval (95% CI) [0.278:1.199]), lower thalamic volume (*p* = 0.002, *d* = 0.727, 95% CI [0.265:1.185]) and higher NLV (*p* = 0.010, *d* = − 0.603, 95% CI [− 1.056: − 0.146]), compared to CP PwMS. As depicted in Fig. 2, increased levels of sNfL and sGFAP were found in CI compared to CP PwMS (*p* = 0.010, *d* = − 0.605, 95% CI [− 1.064: − 0.141];* p* = 0.035, *d* = − 0.492, 95% CI [− 0.947: − 0.035], respectively). No differences were found for cNfL and cGFAP. In a final step, a sensitivity analysis was performed. By only including PwMS who have CSF measures available (*N* = 54), it was checked whether differences between CP and CI PwMS regarding sNfL and sGFAP were still present as confirmation. Although levels of sGFAP were similar between cognitive groups (*p* = 0.088), levels of sNfL were still increased in CI PwMS (mean sNfL = 12.95 ± 7.75 pg/ml) compared to CP PwMS (mean sNfL = 11.03 ± 10.95 pg/ml; *p* = 0.043).

### Associations between fluid and imaging (bio)markers and cognitive functioning

#### Fluid biomarkers and cognitive domains

**Fig. 3:**
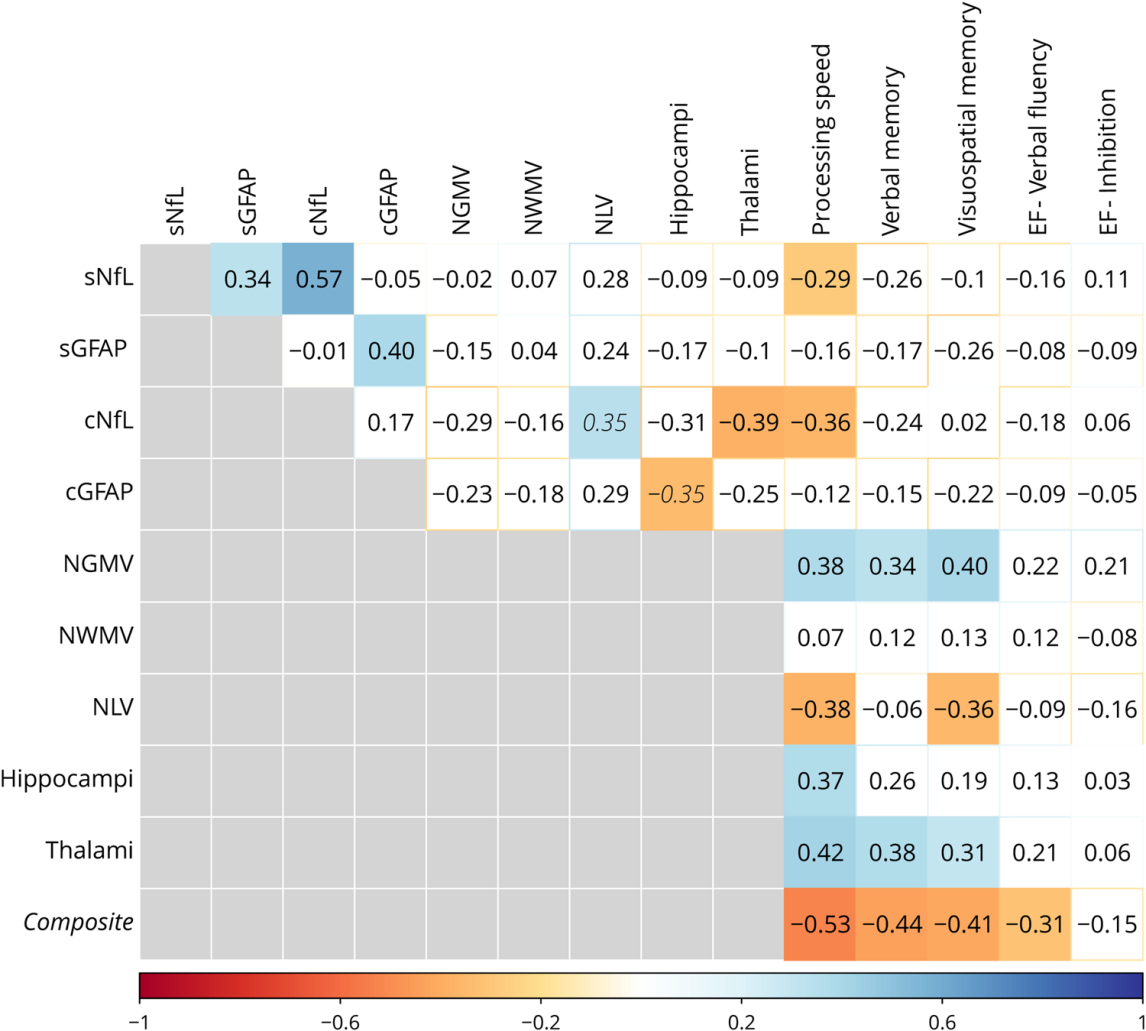
An overview of the correlations between fluid biomarkers, imaging markers and cognitive domains. The correlation coefficient is displayed inside the blocks. Only significant correlations are shown in color (after correction for multiple comparisons; in italic if borderline significant). Correlations between cognitive domains and a post-hoc calculated composite score (a combination of significant predictors (sNfL and NGMV)) is depicted on the bottom row. *sNfL* serum neurofilament light (NfL), *sGFAP* serum glial fibrillary acidic protein (GFAP), *cNfL* CSF NfL, *cGFAP* CSF GFAP, *NGMV* normalized grey matter volume, *NWMV* normalized white matter volume, *NLV* normalized lesion volume, *EF* executive function

As depicted in Fig. 3, reduced processing speed was associated with increased levels of sNfL (*r* = − 0.286, *p* = 0.012) and cNfL (*r* = − 0.364, *p* = 0.007).

#### Imaging markers and cognitive domains

Reduced processing speed was associated with lower NGMV, thalamic and hippocampal volume (range of coefficients: 0.371–0.422), and increased NLV (*r* = − 0.376). Reduced verbal and visuospatial memory were associated with lower NGMV and thalamic volume, with only an association with increased NLV for visuospatial memory. Correlation coefficients are included in Fig. 3.

#### Fluid and imaging (bio)markers

Increased levels of cNfL were associated with reduced thalamic volume (*r* = − 0.389, *p* = 0.004) and borderline increased NLV (*r* = 0.345, *p* = 0.011, Fig. 3). Finally, increased levels of cGFAP were associated with reduced hippocampal volume (*r* = − 0.347, *p* = 0.010), although this finding was borderline significant.

No other correlations between fluid and imaging (bio)markers and cognitive functioning survived correction for multiple comparisons (Fig. 3).

### Prediction of cognitive status

In the first logistic regression model, imaging markers (i.e., NGMV, NLV and thalami), sNfL and sGFAP were included as predictors of cognitive status (*N* = 73). Only NGMV and sNfL were able to predict cognitive status (Table 2). When added to the model, sNfL significantly improved the prediction of cognitive status compared to NGMV alone (sensitivity increased from 70 to 77.5%, whereas the specificity remained 60.6%; *p* = 0.025). The final model, including NGMV and sNfL, resulted in a sensitivity of 77.5%, a specificity of 60.6%, a positive predictive value (PPV) of 70.5%, a negative predictive value (NPV) of 69.0% and an accuracy of 69.9%. In a post-hoc analysis, the independent value of sNfL in the prediction of cognitive status was explored (Table 2). A correct classification of CI PwMS was found in 84.1% PwMS (specificity = 48.5%).

**Table 2:** Results of binary logistic regressions of predicting cognitive status (CP vs. CI)

Models	Predictors	*B*	SE	Wald	*p* value	Odds-ratio [95% CI]
1. Serum and imaging markers						
Step 1^a^	Constant	11.97	4.16	8.27	0.004	
	NGMV	− 0.01	0.05	7.93	0.004*	0.987 [0.977–0.996]
Step 2^b^	Constant	10.91	4.31	6.41	0.011	
	NGMV	− 0.01	0.05	8.18	0.004*	0.986 [0.976–0.996]
	sNfL	2.96	1.32	5.01	0.025*	19.204 [1.443–255.635]
1.1 Post-hoc						
Step 1^c^	Constant	− 1.45	0.74	3.86	0.050	
	sNfL	3.08	1.27	5.88	0.015*	21.847 [1.808–264.044]
2. CSF and imaging markers						
Step 1^d^	Constant	15.14	5.06	8.95	0.003	
	NGMV	− 0.02	0.06	8.71	0.003*	0.983 [0.972–0.994]

*p < 0.05

*CP* cognitively preserved, *CI* cognitively impaired, *B* regression coefficient, *SE* standard error, *Wald* Wald statistic, *95% CI* 95% confidence interval, *sNfL* serum neurofilament light (NfL) in pg/ml, *NGMV* normalized grey matter volume in ml

Model 1: *N* = 73; *R*^*2*^ = 0.190 (Cox & Snell); 0.253 (Nagelkerke)

^a^*X*^*2*^ = 9.49 (1), *p* = 0.002 (step addition: *p* = 0.002)

^b^*X*^*2*^ = 15.34 (2), *p* < 0.001 (step addition: *p* = 0.016)

Model 1.1: In a post-hoc comparison, the significant predictors of model 1 were compared by running a logistic regression on separate predictors

^c^*N* = 77; *R*^*2*^ = 0.09 (Cox & Snell); 0.12 (Nagelkerke); *X*^*2*^ = 7.04 (1), *p* = 0.008 (step addition: *p* = 0.008)

Model 2: *N* = 54; *R*^*2*^ = 0.182 (Cox & Snell); 0.245 (Nagelkerke)

^d^*X*^*2*^ = 10.85 (1), *p* < 0.001 (step addition: *p* < 0.001)

In a second model, imaging markers (i.e., NGMV, NLV and thalami), cNfL and cGFAP were included as predictors of cognitive status (*N* = 54), with only NGMV resulting as significant predictor of cognitive status (Table 2). The final model resulted in a sensitivity of 74.2%, a specificity of 65.2%, a PPV of 74.2%, a NPV of 65.2% and an accuracy of 70.4%. Both models yielded similar explained variances of ~ 25% (Table 2).

### Multimodal marker for cognitive functioning

In a post-hoc analysis, the two significant predictors of cognitive status (NGMV and sNfL) were combined into a composite score as a multimodal marker for cognitive functioning in PwMS. Using the standardized betas as weights, the following formula was applied:

$$\mathrm{Composite}= \, \mathrm{ NGMV } \, (\mathrm{corrected}) \times -0.014 +\mathrm{ sNfL }(\mathrm{corrected}) \times 2.955 + 20.$$

The AUC was larger for the composite score (AUC = 0.751, classification = fair), compared to NGMV (AUC = 0.696, classification = poor-sufficient) and sNfL (AUC = 0.680, classification = poor-sufficient). Classification of cognitive status using the composite score resulted in a sensitivity of 85.0%, a specificity of 57.6%, a PPV of 70.8%, a NPV of 76.0% and an accuracy of 72.6%. A higher composite score was associated with increased performance on multiple cognitive domains: processing speed (*r* = − 0.528, *p* < 0.001), verbal memory (*r* = − 0.436,* p* < 0.001), visuospatial memory (*r* = − 0.411, *p* < 0.001) and EF-verbal fluency (*r* = − 0.314, *p* = 0.008; Fig. 3).

## Discussion

This study investigated the relation of NfL and GFAP measured in serum and CSF and cognitive performance in PwMS presenting with cognitive complaints, and their added predictive value compared to conventional imaging markers. Based on the levels of sNfL and sGFAP we were able to distinguish cognitively preserved from cognitively impaired PwMS, albeit with limited diagnostic accuracy. Increased levels of both serum NfL and GFAP were observed in cognitively impaired PwMS compared to cognitively preserved PwMS. NfL levels (in serum and CSF) were inversely associated with processing speed, indicating that decreased processing speed was associated with increased levels of sNfL and cNfL. No correlations could be detected between GFAP (measured in either serum or CSF) and cognitive functioning in PwMS. Finally, sNfL added unique variance in the prediction of cognitive status on top of NGMV. A composite score of both measures (a multimodal marker) resulted in a fair classification of cognitive status, stressing the need for a multimodal approach when predicting cognitive functioning.

Consistent with previous literature, increased levels of sNfL were found for cognitively impaired PwMS [7, 10, 11]. Furthermore, increased levels of sNfL and cNfL were associated with reduced processing speed. Slowed processing speed, has been hypothesized to be the major driver of cognitive impairment in MS [1], thereby possibly explaining why correlations with this specific domain are more prevalent in studies investigating NfL and cognitive functioning in MS [10, 12]. Yet, mixed results have been reported for increased levels of NfL and the performance in other cognitive domains [11]. Differences in sample size, administered neuropsychological tests, study population (i.e., a focus on newly diagnosed PwMS [12] or SPMS [8], a combination of MS types, or PwMS with mild cognitive impairment [39]) and specific focus on treatment are most likely explaining these differences [40]. In our sample, the distribution of PwMS on DMT at the time of the visit (but also the distribution of PwMS on first-line DMT vs. second-line DMT) was similar between cognitive groups, thereby reducing the likelihood of impacting our findings. Nonetheless, it could have played a role on an individual level as has been shown before [41]. Although it was beyond the scope of current research, the impact of DMTs on cognitive functioning in MS warrants further investigation [1]. Finally, although levels of sGFAP were increased in cognitively impaired PwMS, no correlations between GFAP and the cognitive domains survived correction for multiple comparisons, hereby limiting its potential as a clinical biomarker for cognitive functioning in MS.

Jakimovski et al. demonstrated in two previous studies a relatively weaker correlation between sNfL and cognition [10], compared to correlations between sNfL and MRI outcomes [42]. As potential explanation they put forward the role of adaptive processes to significantly influence the relationships between the released NfL and cognitive test results. Subsequently, PwMS who demonstrate preserved functional connectivity, despite ongoing structural pathology, can maintain high levels of cognitive performance [43]. In the current study, associations between sNfL and imaging markers were absent. However, associations of cNfL and sNfL between both processing speed and between cNfL and imaging markers were comparable in effect size with previous studies, thereby confirming aforementioned difference [10, 42]. Interestingly, in our study, imaging markers displayed a higher number of associations with multiple cognitive domains (not only processing speed, but also verbal and visuospatial memory) compared to fluid NfL and GFAP levels, highlighting that structural pathology was present and related to several cognitive test scores. As fluid biomarkers provide a real-time evaluation of the amount of pathology compared to the less dynamic imaging markers [9], it can be hypothesized that cognitive changes are not resulting from acute disturbances but rather from a more global effect over time on the brain in certain areas.

When added to the model, sNfL improved the prediction of cognitive status compared to NGMV alone. Especially when combining biomarkers, in our case NGMV and sNfL (the “multimodal marker”) a large effect was found for processing speed, whereas medium effects were reported for verbal and visuospatial memory. Even a medium sized effect for EF-verbal fluency was found when using the multimodal marker, which was absent when investigating individual markers. The current study is one of the first studies to combine both neuroimaging and fluid biomarkers of interest to detect cognitive impairment in MS. Investigating the role of a multimodal marker for cognitive functioning in PwMS is of high importance since these different modalities might reflect different aspects of neurodegeneration, which also has been reported in Alzheimer’s disease [44] and recently in MS as well [7, 45]. More specifically, previous studies investigating cross-modal fluid and imaging (bio)markers indeed show an “additive” effect of sNfL compared to cortical thickness [45] or lesion load and grey matter volume [7] in recently diagnosed PwMS. Together with our results, the added effect of sNfL highlights the necessity of using multiple sources of information to create a diagnostic marker for something as highly complex as cognitive performance, but also how these markers of neurodegeneration cannot be used interchangeably.

Nonetheless, clinical interpretation may be optimized when the full prognostic potential of sNfL for cognitive functioning will be evaluated over time, which is an important limitation of the current cross-sectional study design. The inclusion of a control group would have further aided the disentanglement between normal and abnormal levels of fluid and imaging (bio)markers. Also, contrary to measurements in serum, both NfL and GFAP measured in CSF were unable to discriminate between cognitive status. The most plausible explanation for this lack of detecting a difference is the limited power (*N* CSF = 54 versus *N* = 78 for serum). Performing a lumbar puncture is rather invasive and not all PwMS wanted to partake in this procedure. Importantly, without a post-contrast sequence being available in current study protocol, it was not possible to determine whether PwMS had active lesions at the time of evaluation. As a consequence, the investigation of the effect of recent disease activity on serum and CSF levels was limited and could be considered an important avenue for future research. Finally, the inclusion of a clinical, real-life sample is one of the biggest strengths, as the PwMS are reflective of our population at the outpatient clinic with perceived cognitive complaints. At the same time, being a real-life sample is also one of the main limitations. A homogenous sample is often desirable when investigating differences between groups, although data on other types of MS than RRMS is often lacking. Furthermore, given the fact that PwMS visited the outpatient clinic because of cognitive complaints, a slight bias towards cognitive impairment may have been present. The main clinical aim of the outpatient clinic is to investigate whether these complaints (or impairments) are due to MS pathology or, for instance, psychological or social factors (known to influence cognitive performance [46]). Results on PROMS measuring mood, anxiety, fatigue and sleep were, therefore, reported in this manuscript showing similar scores between cognitively preserved and impaired PwMS. Consequently, the impact of these factors on cognition was also considered similar.

In conclusion, we provided novel insights into the relationship between fluid biomarkers of neurodegeneration and their relation to cognitive functioning and conventional imaging measures in PwMS. The main finding of this study is the result that sNfL explains additional variance in cognitive performance on top of NGMV. A novel insight that was further explored in our study was the potential for combining two (bio)markers from a different origin when predicting cognitive status, instead of focusing on single measures of NfL or imaging outcomes. Combining multimodal biomarkers may be the way forward to enable timely identification of cognitive decline in MS.

## Supplementary Information

Below is the link to the electronic supplementary material.

Supplementary file1 (DOCX 21 KB)

## Data Availability

Anonymized data, not published in the article, can be shared upon reasonable request from a qualified investigator.
