# Supplementary material for: A multimodal marker for cognitive functioning in multiple sclerosis: the role of NfL, GFAP and conventional MRI in predicting cognitive functioning in a prospective clinical cohort
Source: J Neurol. 2023 Apr 27;270(8):3851–61. doi: 10.1007/s00415-023-11676-4 (PMC10344976; doi:10.1007/s00415-023-11676-4)
Supplement: Supplementary file 1 — Supplementary file1 (DOCX 21 KB) [file 415_2023_11676_MOESM1_ESM.docx]

**Supplementary materials**

Supplemental methods

**Imaging protocol**

A total of 78 PwMS (~91%) underwent MR scanning on a 3-Tesla whole-body scanner (General Electric Signa-HDxt, Milwaukee, WI, USA), with an 8-channel head coil. The details of the MRI protocol consisted of (1) a 3D-T1 weighted fast spoiled gradient echo sequence for brain volumetry and (2) a 3D fluid-attenuated inversion recovery sequence for white matter lesion detection (see supplemental methods Table 1 for acquisition parameters). Subsequent analyses were performed using FSL5 (<http://fsl.fmrib.ox.ac.uk/>).

**Supplementary Table 1.** Information on acquisition parameters of the sequences included in the imaging protocol.

|  | | **3D-T1 FSPGR**  **sequence** | **3D-FLAIR**  **sequence** |
| --- | --- | --- | --- |
| **Parameters** | |  |  |
|  | Acquisition time | 256s | 224s |
|  | Repetition time (TR) | 8.22ms | 8000ms |
|  | Echo time (TE) | 3.22ms | 128ms |
|  | Inversion time (TI) | 450ms | 2343ms |
|  | Flip angle | 12° | 12° |
|  | Orientation | Sagittal | Sagittal |
|  | Voxel size | 1.0mm | 1.2mm |

*Abbreviations: FSPGR = Fast Spoiled Gradient Echo; FLAIR = Fluid-Attenuated Inversion Recovery*

**Fluid biomarker preprocessing and analysis protocol**

After 30 minutes and within two hours, collected blood and CSF (all between 12.00 and 15.00hs) were centrifuged at 1800*g* for ten minutes and all samples were stored at -20°C for the first 24.00hs and thereafter at -80°C until analysis according to consensus protocols.^1^

Supplemental results

**Supplementary Table 2.** The performance on individual neuropsychological tests

|  | | | **Mean (± standard deviation)** |
| --- | --- | --- | --- |
| **Neuropsychological tests** | | |  |
|  | Stroop | |  |
|  |  | Card I | -0.62 (± 1.41) |
|  |  | Card II | -1.28 (± 1.27) |
|  |  | Interference | -1.32 (± 1.28) |
|  | CVLT-2 | |  |
|  |  | Direct recall | -0.93 (± 1.16) |
|  |  | Delayed recall | -0.93 (± 1.46) |
|  |  | Recognition | -0.53 (± 1.13) |
|  | BVMT-R | |  |
|  |  | Direct recall | -1.05 (± 1.03) |
|  |  | Delayed recall | -1.53 (± 1.85) |
|  |  | Recognition | -0.24 (± 0.94) |
|  | SDMT | |  |
|  |  | Total score | -1.59 (± 1.11) |
|  | COWAT | |  |
|  |  | Total score | -0.94 (± 0.73) |

*Abbreviations: CVLT-2 = California Verbal Learning Test version 2; BVMT-R = Brief Visuospatial Memory Test-Revised; SDMT = Symbol Digit Modalities Test; COWAT = Controlled Oral Word Association Test*

**Supplementary Table 3.** Information on demographics, disease related variables, patient reported outcome measures, imaging markers (in ml) and fluid biomarkers (in pg/ml) displayed for cognitive groups.

|  | | **CP**  **(*N* = 33)** | **CI**  **(*N* = 40)** | ***p*-value** |
| --- | --- | --- | --- | --- |
| **Demographics** | |  |  |  |
|  | Sex (female : male) | 29 : 4 | 24 : 16 | **.008*** |
|  | Age | 48.09 ± 9.02 | 47.20 ± 8.06 | .658 |
|  | Educational level | 6 [5-6] | 6 [5-6] | .972 |
| **Clinical functioning** | |  |  |  |
|  | Disease duration^a^ | 13.66 ± 9.39 | 14.12 ± 8.79 | .830 |
|  | EDSS | 3.5 [2.5–4.0] | 4.0 [3.0–4.5] | **.032*** |
|  | MS Type  (CIS/RRMS/PPMS/SPMS) | (2/24/3/4) | (2/25/1/12) | .146 |
|  | Use of DMT (yes : no) | 17 : 16 | 18 : 22 | .750 |
|  | Type of DMT  (first-line: second-line) | 11 : 5 | 14 : 8 | .999 |
| **Patient reported outcomes** | |  |  |  |
|  | HADS anxiety | 8.31 ± 4.15 | 8.39 ± 4.79 | .940 |
|  | HADS depression | 6.16 ± 3.90 | 7.62 ± 4.48 | .341 |
|  | CIS20-R (fatigue) | 88.39 ± 21.86 | 94.03 ± 17.94 | .240 |
|  | MSNQ-P (cognitive complaints) | 32.11 ± 10.44 | 33.82 ± 7.74 | .465 |
|  | AIS (sleep-related problems) | 6.69 ± 4.83 | 7.62 ± 4.48 | .404 |
| **Imaging markers (ml)^b^** | |  |  |  |
|  | NGMV | 805.00 ± 63.95 | 753.25 ± 56.95 | **.002*** |
|  | NWMV | 683.27 ± 46.69 | 668.58 ± 48.48 | .194 |
|  | NLV^c^ | 22.14 ± 21.10 | 35.25 ± 27.85 | **.007*** |
|  | Hippocampi | 9.36 ± 1.12 | 8.55 ± 1.38 | .086 |
|  | Thalami | 19.29 ± 2.34 | 17.13 ± 2.85 | **.004*** |
| **Fluid biomarkers (pg/ml)** | |  |  |  |
|  | sNfL^c^ | 8.45 [5.19–12.67] | 10.33 [8.37–13.94] | **.021*** |
|  | sGFAP^a^ | 103.37 [79.59–147.88] | 124.77 [88.48–181.63] | .071 |
|  | cNfL^c^ | 546.99 [336.23–736.64] | 579.74 [502.50–1109.46] | .134 |
|  | cGFAP^a^ | 7360.16 [5100.24–8887.99] | 8039.92 [6818.75–9307.84] | .086 |

Displayed are the mean and standard deviation of continuous variables, the median and interquartile range of ordinal or non-normally distributed data. Imaging markers and fluid biomarkers were corrected for age and sex (if appropriate) before tested. ^a^Variable was square root-transformed before tested. ^b^All volumes were normalized using the V-scaling factor. ^c^Variable was log-transformed before tested.

*Abbreviations*: *CP = cognitively preserved; CI = cognitively impaired; EDSS = Expanded Disability Status Scale; CIS = clinically isolated syndrome; RRMS = relapsing remitting MS; PPMS = primary progressive MS; SPMS = secondary progressive MS; UN = Unknown; DMT = Disease-modifying Therapy; HADS = Hospital Anxiety and Depression Scale; CIS20-R = Checklist Individual Strength 20 – Revised; MSNQ = MS Neuropsychological Questionnaire. AIS = Athens Insomnia Scale; NGMV = normalized grey matter volume; NWMV = normalized white matter volume; NLV = normalized lesion volume; sNfL = serum neurofilament light (NfL); sGFAP = serum glial fibrillary acidic protein (GFAP); cNfL = CSF NfL; cGFAP = CSF GFAP.*

**References**

1. Teunissen C, Petzold A, Bennett JL, et al. A consensus protocol for the standardization of cerebrospinal fluid collection and biobanking. Neurology 2009;73:1914-1922.
